# Activation of specific bitter taste receptors by olive oil phenolics and secoiridoids

**DOI:** 10.1038/s41598-021-01752-y

**Published:** 2021-11-16

**Authors:** Meng Cui, Bohan Chen, Keman Xu, Aimilia Rigakou, Panagiotis Diamantakos, Eleni Melliou, Diomedes E. Logothetis, Prokopios Magiatis

**Affiliations:** 1 Department of Pharmaceutical Sciences, School of Pharmacy, Bouvé College of Health Sciences, Northeastern University, Boston, MA 02115 USA; 2 Laboratory of Pharmacognosy and Natural Products Chemistry, Department of Pharmacy, National and Kapodistrian University of Athens, Panepistimiopolis Zografou, 15771 Athens, Greece; 3 Chemistry and Chemical Biology, College of Science, Northeastern University, Boston, MA 02115 USA; 4 Center for Drug Discovery, Northeastern University, Boston, MA 02115 USA

**Keywords:** Chemical biology, Natural products, Small molecules, Target identification

## Abstract

Extra-virgin olive oil (EVOO) is a critical component of the Mediterranean diet, which has been found beneficial to human health. Bitterness is often positively associated with the presence of phenolic compounds in EVOO. There are twenty-five bitter taste receptors (TAS2Rs) in humans, each of which responds to specific bitter tastants. The identity of phenolic compounds and the bitter taste receptors they stimulate remain unknown. In this study, we isolated 12 phenolic and secoiridoid compounds from the olive fruit and the oil extracted from it, and tested their ability to stimulate bitter taste receptor activity, using a calcium mobilization functional assay. Our results showed that seven out of twelve studied compounds activated TAS2R8, and five of them activated TAS2R1, TAS2R8, and TAS2R14. The phenolic compounds oleuropein aglycon and ligstroside aglycon were the most potent bitter tastants in olive oil. TAS2R1 and TAS2R8 were the major bitter taste receptors activated most potently by these phenolic compounds. The results obtained here could be utilized to predict and control the bitterness of olive oil based on the concentration of specific bitter phenolics produced during the milling process of olives.

## Introduction

**Figure 1:**
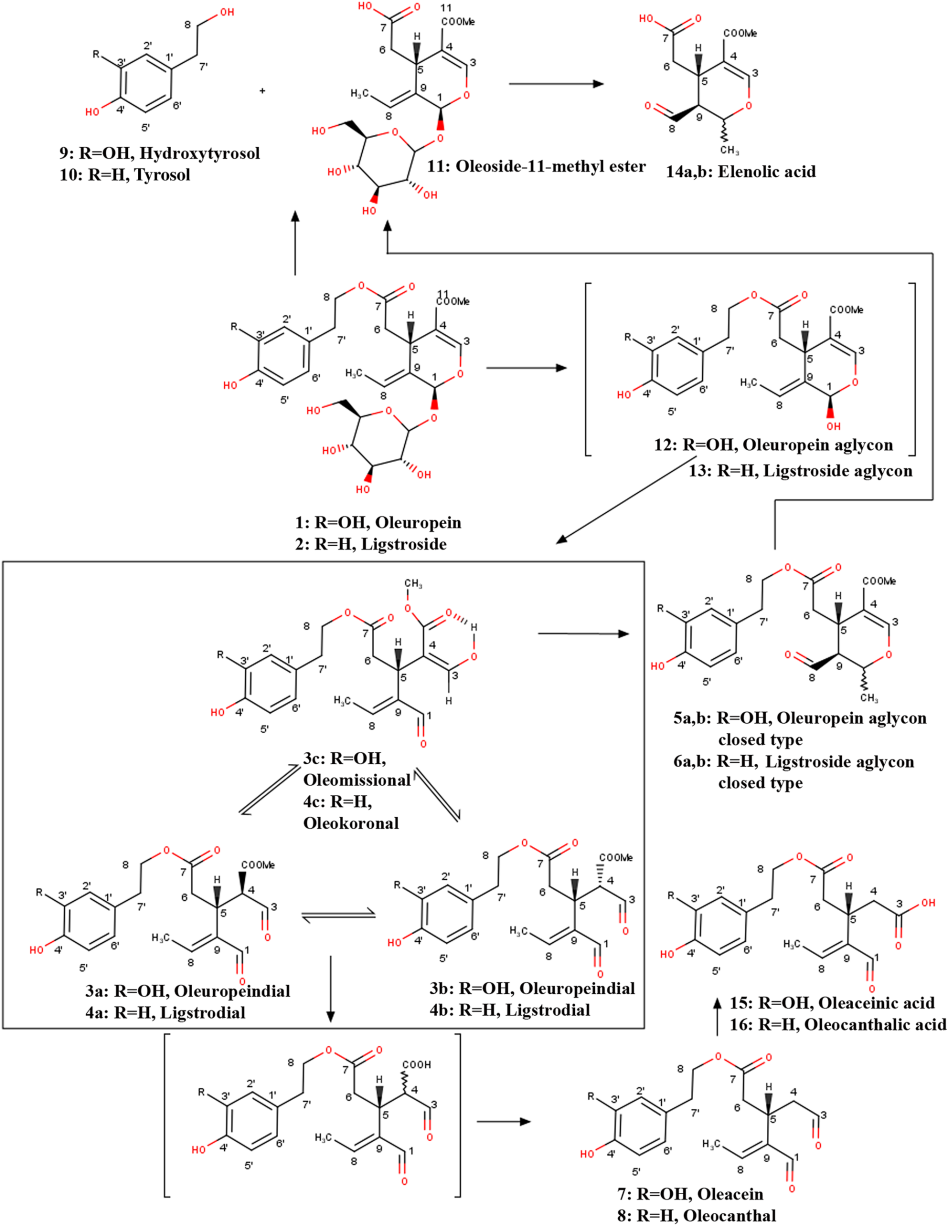
The structures of the studied phenolic compounds from olives and olive oil and their biosynthetic relationships. Oleuropein (1), ligstroside (2), oleuropeindial (3a,b), oleomissional (3c), ligstrodial (4a,b), oleokoronal (4c), oleuropein aglycon closed type (5a,b), ligstroside aglycon closed type (6a,b), oleacein (7), oleocanthal (8), hydroxytyrosol (9), tyrosol (10), oleoside-11-methyl ester (11), oleuropein aglycon (unstable form) (12), ligstroside aglycon (unstable form) (13), elenolic acid (14a,b), oleaceinic acid (15), oleocanthalic acid (16). a,b correspond to the two possible stereoisomers of the molecule.

Extra-virgin olive oil (EVOO) is a critical component of the Mediterranean diet, which is mostly plant-based and includes a high consumption of cereals, vegetables, and fruits with a lower consumption of red meat and sweets. This diet has been found beneficial to human health by reducing the risk for cardiovascular diseases, type 2 diabetes and generally metabolic syndrome, malignancies such as breast and gastric cancer, as well as depression, and cognitive impairment^1,2^. EVOO has a significant role in decreasing cardiovascular disease displaying anti-inflammatory and antioxidant properties. EVOO contains numerous substances that have a beneficial role in human health, including phenols, which are produced at the malaxation step during production of olive oil, starting from oleuropein (**1**) and ligstroside (**2**) (Fig. 1). Oleuropein aglycons (**3a**,**3b**,**3c**,**5**), ligstroside aglycons (**4a**,**4b**,**4c**,**6**), oleacein (**7**) and especially oleocanthal (**8**) are the most abundant phenols in EVOO^3^ (Fig. 1). Oleocanthal has been suggested to act as a neuroprotective agent in Alzheimer disease^4–9^ and to act as an anti-inflammatory and an anti-cancer agent^10–12^. Oleacein is a potent antioxidant agent with anti-inflammatory activities^13^ helpful against atherosclerosis^14^, as an antiaging factor^15^ and as a neuroprotective agent^16^. Ligstroside aglycon has a great effect against cell migration, which suggests it can serve as a potent agent against malignancies, such as breast cancer^17,18^. Oleuropein aglycon has been reported to display activities against Alzheimer disease^19,20^, and breast cancer^18^. Bitterness is often positively associated with the presence of phenolic compounds in EVOO. Oleuropein and ligstroside derivatives are major substances responsible for the bitter taste of EVOO. Although most of the studies regarding the health benefits of the phenolic compounds of EVOO have been performed in vitro or in vivo and less in humans, users are encouraged to consume olive oil high in phenolic compound content. However, excessive bitterness of olive oil could alienate certain consumers sensitive to the taste. Thus, identifying the main contributors of bitter taste and controlling their concentration during production would ensure the benefits they have, while maintaining the palatability of the olive oil for most consumers.

Olive oil is classified as extra virgin, virgin or lampante, depending on several chemical and organoleptic criteria^21^. The organoleptic characterization of olive oil includes positive and negative taste attributes. The positive taste attributes are fruity, pungent and bitter. European Union (EU) legislation does not classify olive oil as extra virgin or virgin if the fruity attribute is not detected. The bitter and pungent taste attributes are positively evaluated by trained tasters although in the past many consumers were erroneously considering these attributes as defects^22,23^. The pungent taste, which is characteristic of early harvest olive oils produced by unripe olives, is a burning sensation resulting from the action of certain phenolic substances mainly perceived in the pharynx^24,25^, and gradually disappearing after tasting. Even though this property of olive oil produced by green unripe olives was associated with its healthy properties ~ 2000 years ago by the Greek physician Dioscorides, it has not been until recently that its biological basis has begun to unravel. Oleocanthal, a highly bioactive and health protecting compound more abundant in early harvest olive oils^26^, activates TRPA1 receptor/channels^25^ contributing to the irritation of the pharynx^24^.

The bitter attribute is also characteristic of olive oils obtained by green olives or olives turning color. It is also considered to be related with the phenolic substances of olive oil and bitter taste perceived in the tongue. Although it is known that olive oils with low phenolic content have reduced bitterness, it is neither known which phenolic compounds are bitter and to what extent nor through which bitter receptors they act.

The importance of the sense of taste for humans and other organisms lies in the detection and evaluation of food quality. In mammals at least five taste qualities can be detected and discriminated that include sweet, bitter, sour, salty and umami^27^. The sense of taste bears a significant role in human food selection, nutrition and health. Bitter taste perception involves detection among a large number of structurally divergent bitter compounds^28–30^. Many naturally poisonous substances taste bitter and elicit aversive responses in humans and other mammalian species, suggesting that bitter transduction is a key defense mechanism in the avoidance of harmful substances^30,31^. However, there are also plenty of bitter tasting food products with clear benefits, such as olive oil, coffee, vegetables and medicines, such as ancient Chinese herbal medicines^32^. In addition, extraoral expression of bitter taste receptor throughout the body mediate diverse nontasting roles through various mechanisms^33–35^.

Especially for olive oil the EU has adopted a legislation^36^ since 2012 permitting the use of health claim related to protection of blood lipid oxidation to all olive oils having more than 5 mg phenolics per 20 g. The exact definition concerns the phenolic ingredients that are considered derivatives of hydroxytyrosol, tyrosol or belonging to the oleuropein complex (**1–10**) (see Fig. 1) and are potentially bitter. Therefore, it is highly desirable to characterize and control bitter taste perception to ensure palatability of specific beneficial food products and medicines^37^.

In humans, there are 25 functional G protein-coupled receptors (GPCRs) that belong to the TAS2R gene family mediating bitter taste^38^. TAS2R genes, each coding for about 290–330 amino acid proteins, are structurally diverse. Across the TAS2R gene family there is approximately 17–90% sequence identity, which suggests that different family members may recognize bitter tastants with diverse structures^39^. Yet, sequence differences do not allow prediction of bitter tastants with diverse structures, as closely related receptors can show very different agonist profiles^40^, identical agonists can signal through very diverse bitter taste receptors^41^ and subtle differences in the binding pocket can be predictive for agonist specificities^42^. Four TAS2Rs of the 25 remain to be deorphanized: TAS2R19, TAS2R42, TAS2R45, and TAS2R60^43^. TAS2Rs couple to G_i_-type G proteins, most often gustducin, but also transducin and G_αi2_ in some taste receptor cells (TRCs). Upon TAS2R activation, and dissociation of the heterotrimeric G protein (e.g. G_α_ gustducin/β_3_/γ_13_), the β_3_/γ_13_ complex released from activated α-gustducin stimulates PLCβ_2_ to generate inositol trisphosphate (IP_3_) and diacylglycerol (DAG). IP_3_ leads to release of Ca^2+^ from internal stores. Calcium ions in turn activate TRPM5 channels resulting in changes of membrane potential leading to cell depolarization, generation of action potentials and neurotransmitter release^28,43^. G_α_ gustducin stimulates phosphodiesterase (PDE) to hydrolyze cAMP that exerts a modulatory role compared to the primary signaling role elicited by Ca^2+^; the decreased cAMP may disinhibit cyclic nucleotide-inhibited channels and also elevate intracellular Ca^2+^^28^.

In recent years, an enormous progress in the deorphanization of hTAS2R genes has been achieved by functional heterologous expression of these receptors in mammalian cell lines. For efficient functional expression at the cell surface, amino terminal ‘export-tags’, such as the amino termini of the rat somatostatin receptor subtype 3 or the bovine rhodopsin, were linked to receptor cDNAs^44,45^. Co-expression of TAS2Rs with a G protein chimera (G_α16-gus44_), in which the last 44 amino acids in the C-terminal end of G_α16_ are replaced by the corresponding residues of gustducin, allow coupling of G_α16_ to TAS2Rs and signaling towards Ca^2+^ mobilization upon receptor activation. Such constructs have been used to test TAS2R function^46^.

The current trend in the olive market is the promotion of olive oils with high phenolic content based on their health-protecting properties, however their increased bitterness is not favorable especially for non-Mediterranean consumers who are not used to this taste. Therefore, it would be very useful for the olive oil industry to know the identity of the phenolic compounds mainly responsible for the bitter taste in order to find ways to control their concentration while maintaining the phenolic content required to qualify for the health claim as described by the EU legislation^36^.

## Materials and methods

### Materials

1,10-phenanthroline, andrographolide, chloramphenicol, chloroquine, cromolyn, denatonium benzoate, flufenamic acid, procainamide, strychnine, salicin, 6-n-propylthiouracil (PROP), cucurbitacin E, ranitidine, aristolochic acid, humulone were obtained from Sigma. Trp-Trp-Trp was obtained from BACHEM. Oxyphenonium was obtained from Santa Cruz Biotechnology. Olive phenolic compounds, oleuropein^47^ (1), oleomissional/oleuropeindials mixture^48^ (3a,b,c), oleuropein aglycon closed-type (isomer A) (5a), oleuropein aglycon closed-type (isomer B) (5b), ligstroside aglycon closed type^49^ (6a,b), oleacein (7), oleocanthal (8), hydroxytyrosol (9), tyrosol (10), oleoside-11-methylester^50^ (11), elenolic acid^51^ (14a,b), and oleocanthalic acid^52^ (16) (Fig. 1) were isolated in the Magiatis lab from olives or olive oil using chromatographic methods as previously described^26,47–52^, and their purity was > 95% as measured by qNMR. The olives used in this study were collected from olive trees of the University of Athens campus with permission for research use. All local, national or international guidelines and legislation were adhered to carrying out the present study. Fetal bovine serum, Gibco HBSS buffer, Gibco 1 M HEPES (4-(2-hydroxyethyl)-1-piperazineethanesulfonic acid) buffer, Opti-MEM Reduced Serum Medium, and GlutaMAX Supplement culture medium were obtained from Life Technologies. 96-well poly-D-lysine plates were purchased from Corning life sciences.

### Bitter taste receptor TAS2R constructs

25 human TAS2R genes were subcloned into the pcDNA 3.1 vector, as previously descripted in the literature^44,53^. The first 45 amino acids of the rat somatostatin type 3 receptor, which serves as a cell-surface-targeting signal, were added to the N-termini of the TAS2Rs to target them more efficiently to the cell surface. Herpes simplex virus (HSV) glycoprotein D epitopes were added to the carboxy termini of the receptors for immunocytochemistry^44^. All constructs were confirmed by DNA sequencing.

### Calcium mobilization functional assay

HEK293E cells were cultured at 37 °C in DMEM (Thermo Fisher Scientific), supplemented with 10% fetal bovine serum (FBS) and 1% Penicillin Streptomycin. The cells were seeded onto 96-well plates (Clear Bottom Black Polystyrene Poly-D-Lysine Coated Microplates, Corning life sciences) at a density of 50,000 (in 100 μl DMEM) per well, and cultured at 37 °C for overnight. On the second day following seeding, the medium was changed to OPTI-MEM supplemented with 5% FBS (filtered) before co-transfection using Polyethylenimine (PEI) (1.6 μg/well) with plasmid DNAs encoding TAS2Rs (0.1 μg/well), Gα16-gust44 (0.06 μg/well) and the GCaMP calcium sensor (0.035 μg/well). After 24 h, the medium was changed to fresh OPTI-MEM supplemented with 5% FBS (filtered) and 0.1% Plasmocin (InvivoGen). After an additional 24 h, the cells were washed with Hank’s Buffered Salt Solution (HBSS) supplemented with 20 mM HEPES (HBSSH). The plates were then placed into a FlexStation 3 system (Molecular Devices) to monitor fluorescence changes (excitation, 488 nm; emission, 525 nm; cutoff, 515 nm) after the addition of 50 μl of HBSSH supplemented with 2 × testing of compounds (tested compounds were first diluted in DMSO (100–1000 mM)). For each trace, a compound was added at 30 s following the start of the read. Readings continued to be taken for an additional 150 s, and data were collected every 2 s. Calcium mobilization in response to test compounds was quantified as the percentage of change (peak fluorescence—baseline fluorescence level, denoted as ΔF) from its baseline fluorescence level (denoted as F). Data from single dose experiments were collected as duplicates. For the dose–response curves, data were collected as triplicates. Relative data were obtained by normalizing to one of the strongest signals (e.g. TAS2R8 activation obtained with oleuropein aglycon B (100%)). For EC_50_ calculations, plots were prepared by nonlinear regression three parameter dose–response curve equation in GraphPad. Statistics analysis was performed by using one-way ANOVA Tukey's multiple comparison test in GraphPad. Data represent mean ± SD.

## Results

### Functional assay of bitter taste receptors

**Figure 2:**
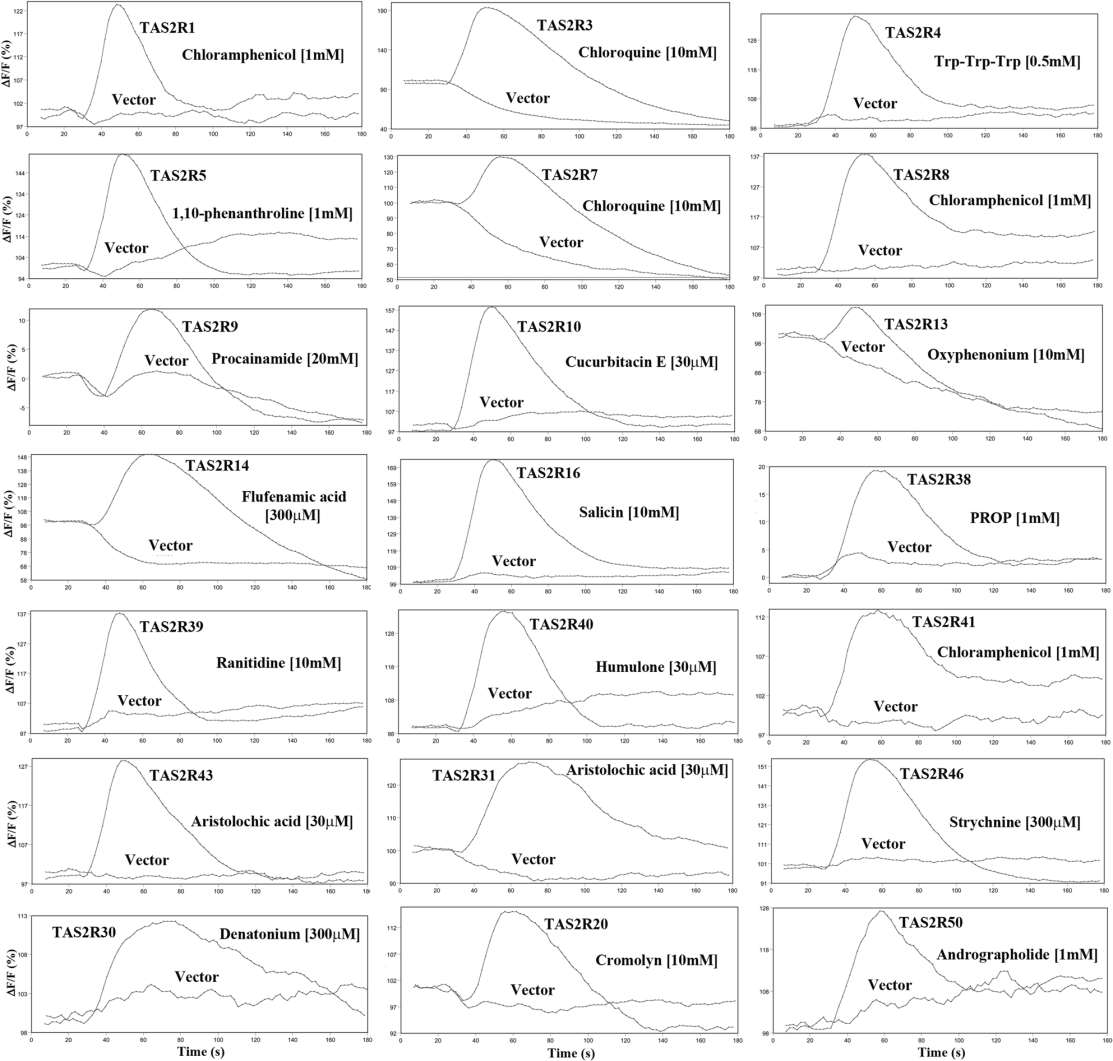
Functional results of deorphanized TAS2Rs (TAS2Rs) response to their representative bitter compounds, comparing with empty pcDNA vector as a negative control. The TAS2R DNAs, G16-gust44, and GCaMP calcium sensor were co-expressed in HEK293E cells. The fluorescence signal changes were measured by adding representative bitter compounds to the corresponding TAS2Rs, using a FlexStation 3 microplate reader (96 well plate).

To assess the function of newly made TAS2R cDNA constructs, we transiently expressed TAS2Rs together with cDNA of the G_α16-gust44_ protein chimera in HEK293E cells. The receptor transporting proteins (RTP3 and RTP4) were also co-expressed with TAS2R1 and TAS2R20 to enhance receptor expression^54^. The cognate bitter tasting compounds of TAS2Rs were selected from the BitterDB database (http://bitterdb.agri.huji.ac.il/bitterdb/). We performed calcium mobilization assay experiments to test the function of the TAS2R receptors activated by their cognate bitter taste compounds. Functional results from 21 deorphanized TAS2Rs are shown in Fig. 2, where the receptors responded to their corresponding representative bitter compounds compared to the negative control (each receptor was replaced by the empty pcDNA vector).

The results showed that TAS2R1, 8, and 41 receptors were activated by chloramphenicol [1 mM]^43,55–57^; TAS2R3 and 7 were activated by chloroquine [10 mM]^58^; TAS2R4 was activated by the Trp-Trp-Trp peptide^59^; TAS2R5 was activated by 1,10-phenanthroline [1 mM]^60^; TAS2R9 was activated by procainamide [20 mM]^61^; TAS2R10 was activated by cucurbitacin E [30 µM]^41^; TAS2R13 was activated by oxyphenonium [10 mM]^62^; TAS2R14 was activated by flufenamic acid [0.3 mM]^56^; TAS2R16 was activated by salicin [10 mM]^63^; TAS2R38 was activated by PROP [1 mM]^64^; TAS2R39 was activated by ranitidine [10 mM]^62,65^; TAS2R40 was activated by humulone [30 µM]^66^; TAS2R43, and TAS2R31 were activated by aristolochic acid [30 µM]^40^; TAS2R46 was activated by strychnine [300 µM]^41^; TAS2R30 was activated by denatonium [300 µM]^56^; TAS2R20 was activated by cromolyn [10 mM]^56^; and TAS2R50 was activated by andrographolide [1 mM]^67^. In contrast, the negative controls, which the TAS2Rs were replaced by the empty vector, lacked responses to these bitter compounds (Fig. 2).

### Bitter responses of olive oil phenolics and secoiridoids

**Figure 3:**
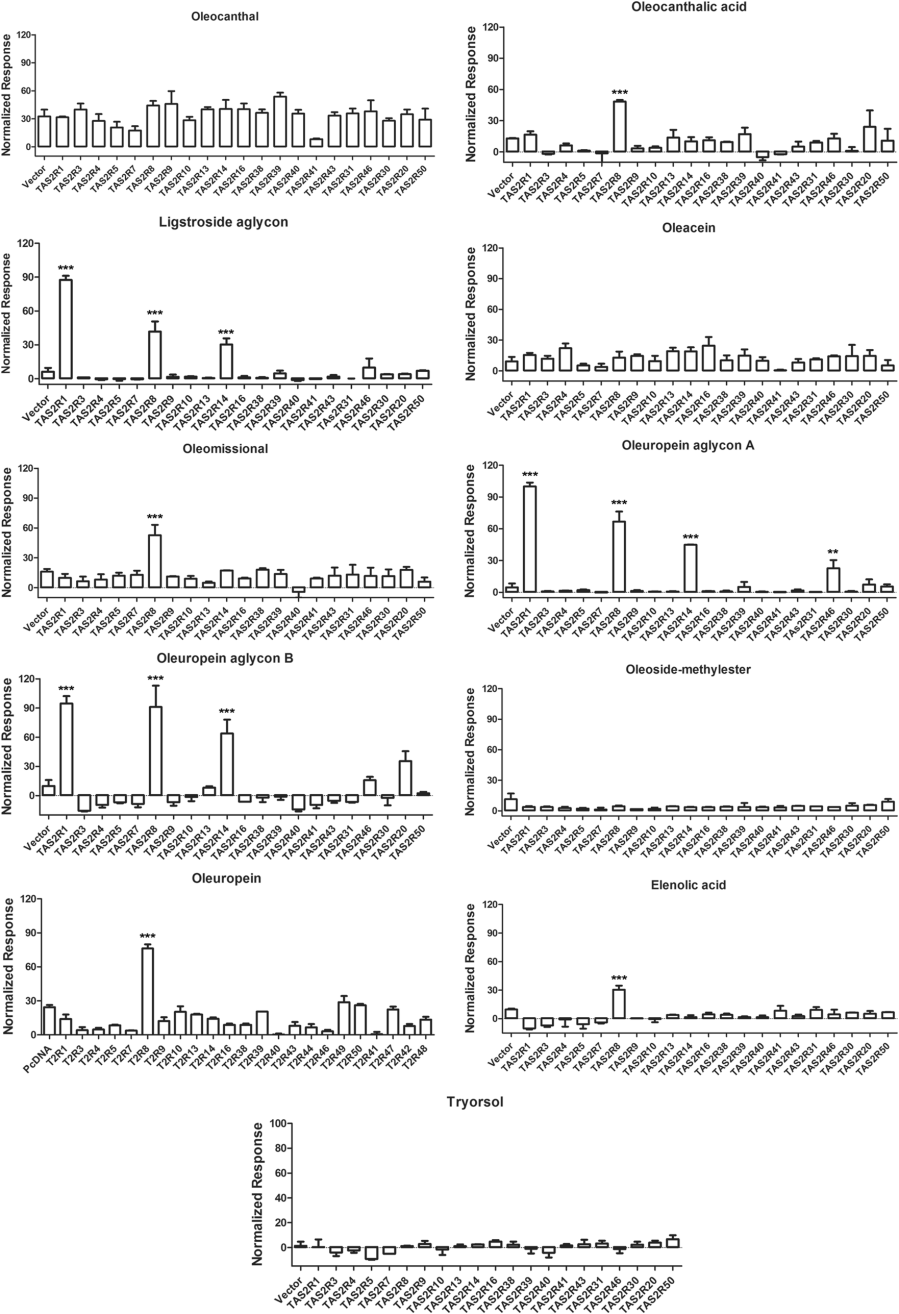
Phenolic compound bitter taste activity tests on hTAS2Rs. Relative fluorescence signals caused by phenolic compound activation of the receptors and the empty vector as a negative control. The concentration of the compounds used was [300 µM], except for Ligstroside aglycon and Oleuropein aglycon [200 µM], oleuropein that was [3 mM]. The asterisks indicate significant differences tested by one-way ANOVA (***p < 0.0001) (N = 2). Data represent mean ± SD.

**Figure 4:**
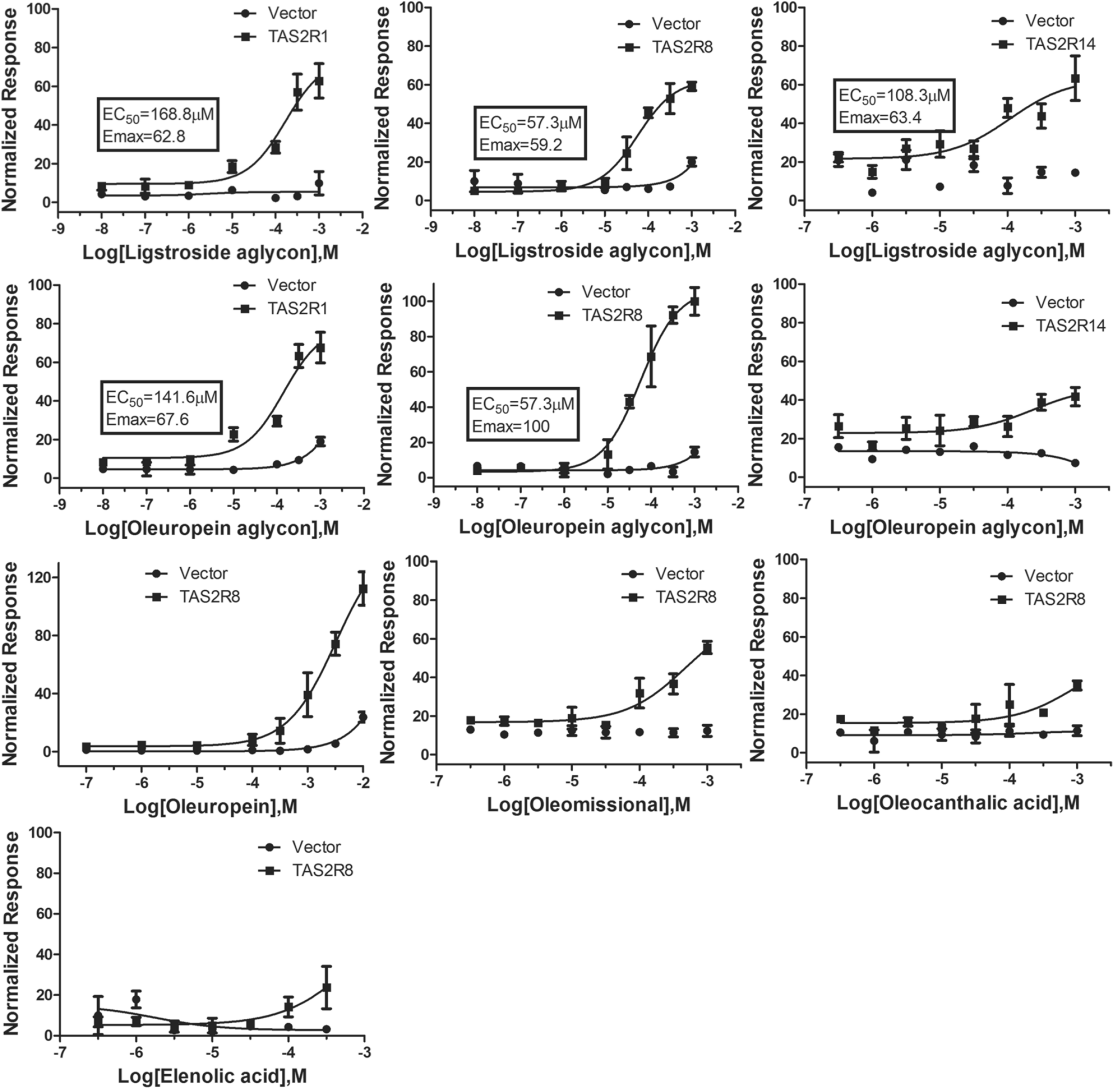
Dose–response curves of olive oil phenolics for bitter taste receptors, TAS2R1, TAS2R8 and TAS2R14. The fluorescence signals were normalized to the highest response of TAS2R8 to oleuropein aglycon (100%) (N = 3). Data represent mean ± SD.

To characterize the bitterness of olive oil phenolic compounds, we performed calcium mobilization experiments. The 25 bitter taste receptors, G protein chimera (G_α16-gus44_), and the GCaMP calcium sensor^68^ were transiently expressed in HEK293E cells. Twelve olive oil phenolic compounds (Fig. 1) were tested for bitter taster receptor activation (Fig. 3). Oleocanthal, oleacein, oleoside-methylester, and tyrosol did not activate any TAS2Rs. In contrast, oleocanthalic acid, oleomissional, oleuropein and elenolic acid activated significantly the TAS2R8 receptor over background. Ligstroside aglycon, and Oleuropein aglycon activated TAS2R1, TAS2R8 and TAS2R14 receptors. To validate the single dose scanning results, we performed concentration–response experiments for all the compounds toward receptors TAS2R1, TAS2R8, and TAS2R14 that produced the most significant responses (Fig. 4). The results showed that of the three receptors, the TAS2R8 receptor yielded the highest and most potent response to oleuropein aglycon, which we called 100% and normalized responses of the other phenolic compounds relative to that (Emax = 100, EC_50_ = 57.3 µM). Ligstroside aglycon was equipotent but less efficacious than oleuropein aglycon (Emax = 59.2, EC_50_ = 57.3 µM). Moderate and less potent activation was seen by the action of these compounds on the TAS2R1 and TAS2R14 [TAS2R1: ligstroside aglycon (Emax = 62.8, EC_50_ = 168.8 µM), oleuropein aglycon (Emax = 67.6, EC_50_ = 141.6 µM); TAS2R14: ligstroside aglycon (Emax = 63.4, EC_50_ = 108.3 µM), and even less potent and efficacious for oleuropein aglycon. The TAS2R8 could also be activated weakly and less potently by oleocanthalic acid, oleomissional and elenolic acid. Interestingly, oleuropein showed high efficacy on TAS2R8 but very low potency and did not reach saturation in the maximal concentration attempted (10 mM).

## Discussion

The current work provides important results concerning the structure–activity relationships of the secoiridoids-phenolics of the olive fruit and oil. Since the discovery of its structure in 1960^69^, oleuropein (**1**) has been thought to be the main source of bitterness of the olive fruit considering its removal by hydrolysis and/or oxidation to make the olive fruit edible. Herein, we show that the derivatives of oleuropein hydrolysis present stronger or weaker potency of bitter receptor activation depending on their structural characteristics.

The oleuropein molecule (and similarly the ligstroside molecule) has three functional groups that can act as targets of hydrolysis. The first one is the ester group attaching the hydroxytyrosol (**9**) (or tyrosol (**10**)) moiety to the secoiridoid skeleton (see Fig. 1). Hydrolysis of this group leads to oleoside-11-methyl ester (**11**) and hydroxytyrosol (or tyrosol) both compounds that failed to activate any bitter receptor (at least up to the concentration tested). The second functional group is the glycosidic bond between glucose and the secoiridoid skeleton. Removal of the glucose moiety from oleuropein (or ligstroside) leads to an unstable aglycon form (**12** or **13**) that is spontaneously transformed to a mixture of two diastereomeric oleuropeindials (**3a,b**) (or ligstrodials (**4a,b**)), which are in equilibrium with the corresponding enolic form named oleomissional (**3c**) (or oleokoronal (**4c**)), all of them having an open ring structure. This mixture of the three compounds in equilibrium can be rearranged to a stable closed ring form (**5a,b** or **6a,b**), existing in two possible diastereomeric forms, with mainly one of them naturally predominating in olive oil. This rearrangement has been found to be dependent on the pH of the medium. Interestingly, the loss of glucose led to compounds with increased potency for activation of bitter receptors. The oleomissional equilibrium mixture (**3a,b,c**) was more potent than oleuropein (**1**) and the closed ring forms (**5a,b**) were almost 50 times more potent than oleuropein and significantly more potent than all other tested compounds. The closed ring form of ligstroside aglycon (**6a**) showed similar but slightly less potent activity than the oleuropein aglycon closed ring forms (**5a,b**), showing that the substitution pattern of the aromatic ring is not crucial for the activity. Comparison between the two closed ring isomers of oleuropein aglycon **5a** and **5b** showed that the stereochemistry of the rearranged secoiridoid ring does not have a very crucial role either. Interestingly, simultaneous removal of the hydroxytyrosol and the glucose moiety from the molecule of oleuropein leading to elenolic acid (**14a,b**) showed higher potency than oleuropein but lower than oleuropein aglycon (**5a,b**), suggesting that the presence of the aromatic ring is important for the activity. Comparison between elenolic acid (**14a,b**) and oleoside methyl ester (**11**) showed that the removal of glucose is also very important for the potency of activation of the bitter receptors.

The final part of the molecule that can be a target of hydrolysis is the methyl ester at C-11 of oleuropein or ligstroside. This ester is removed during malaxation for the production of olive oil by specific esterases leading to unstable carboxy derivatives that are spontaneously decarboxylated. This reaction happening to oleomissional (**3c**) or oleokoronal (**4c**) leads correspondingly to oleacein (**7**) or oleocanthal (**8**). Interestingly, both compounds did not show any activation of bitter receptors. However, oxidation of oleocanthal (**8**) to oleocanthalic acid (**16**) increased the activity of the bitter receptors. Oleocanthalic acid (and its counterpart oleaceinic acid (**15**)) are formed during aging of olive oil and this could explain the retention of some bitterness in aged olive oils.

The above structure–activity information can be summarized in that the most important structural requirement for activation of bitter taste receptors by olive secoiridoid phenolics is the absence of the glucose moiety and the presence of a rearranged closed ring combined with an aromatic group. The open ring forms (**3a,b,c**) retain some potency, but this is much lower compared to those with closed ring forms (**5a,b** or **6a,b**) and is almost completely lost when the open ring forms are decarboxymethylated (**7** or **8**). However, the decarboxymethylated derivatives (oleocanthal and oleacein) are known as TRPA1 receptor activators leading to the pungent effect of olive oil and this is also a very important organoleptic attribute of olive oil. It will be interesting in the future to compare the actions of these phenolic compounds on atomic resolution structures of these protein targets in order to understand their specific binding and actions.

Concerning potential practical applications of the above observations, it is obvious that production of olive oils with reduced bitterness should be performed in such a way that would reduce the content of the closed or open ring aglycons. We have recently shown that the time and temperature of malaxation can play a crucial role towards this direction^70^. More specifically, we have shown that for specific varieties (e.g. Koroneiki or Athinolia) the condition of malaxation (45–60 min at 28 °C) can lead to increased transformation of oleuropein (**3a,b,c**) and ligstroside aglycons (**4a,b,c**) to oleacein (**7**) and oleocanthal (**8**) respectively. In contrast, very short time (15 min) or low temperature (< 23 °C) of malaxation can reduce the oleocanthal or oleacein content in favor of the more bitter aglycons but may also lead to a reduction in oil yield and oil overall flavor.

Another very interesting observation is that malaxation under acidic conditions (e.g. by adding citric acid in the malaxation pulp) can block the rearrangement of the open ring aglycons (**3a,b,c** or **4a,b,c**) to the closed ring ones (**5a,b** or **6a,b**) and can also block the decarboxymethylation reaction^71^. This could serve as an alternative way to produce olive oil with reduced bitterness and pungency.

Regarding the olive debittering process it should be noted that the real target should not only be the removal of oleuropein, as traditionally considered, but also the reduction of the aglycons concentration, achieved by the combined action of glucosidase and esterases. Oleuropein has indeed low potency towards the receptor TAS2R8 but we hypothesize that when we chew on a raw olive fruit the intolerable bitter sensation is mainly triggered by the spontaneous transformation of oleuropein to the aglycon forms and not only by oleuropein itself. Oleuropein and oleuropein glucosidase are found in separated cellular compartments^72^ and can come in contact only when the olive fruit cells are damaged, as it happens when we chew on a raw olive fruit or when the olive fruit is crushed for the production of olive oil.

In conclusion, we isolated and tested bitter taste receptor responses of 12 phenolic compounds from olive oil using the calcium mobilization functional assay. Seven out of twelve phenolics activated TAS2R8, and five of them activated TAS2R1, TAS2R8, and TAS2R14 (Table 1). The phenolic compounds ligstroside aglycon and oleuropein aglycon were the most potent bitter tastants in olive oil. TAS2R1 and TAS2R8 were the major bitter taste receptors responding to phenolic compounds, while TAS2R14 showed a mild response to some of the phenolics. Oleuropein activated TAS2R8 only, but the potency was much lower (50-fold) compared to the algycons. Oleocanthalic acid, oleomissinal, elenolic acid activated TAS2R8 with lower potency. The four phenolics tested, oleacein, oleoside-methylester, oleocanthal, tyrosol failed to activate any TAS2Rs. Organoleptic studies with controlled concentrations of our identified bitter tasting compounds should follow this work to test whether sensory data agree with the TAS2R data presented in the present study. These results could be utilized to control the concentration of bitter phenolics (through the milling process of olives during olive oil production) and thus the intensity of the bitter taste of EVOO to palatable levels.

**Table1:** Summary of the compounds from olive oil and bitter taste receptor activation (*bolded numbers in parentheses identify each compound with the number used in Fig. **1*).

	hTAS2R1	hTAS2R8	hTAS2R14
Ligstroside aglycon (**6a,b**)	+	+	+
Oleuropein (**1**)	−	+	−
Oleacein (**7**)	−	−	−
Oleoside-methylester (**11**)	−	−	−
Oleuropein aglycon closed-type (**5a**)	+	+	+
Oleuropein aglycon (**5b**)	+	+	+
Oleocanthal (**8**)	−	−	−
Oleocanthalic acid (**16**)	−	+	−
Oleomissional (**3a**,**3b**,**3c**)	−	+	−
Elenolic acid (**14a,b**)	−	+	−
Hydroxytyrosol (**9**)	−	−	−
Tyrosol (**10**)	−	−	−

## Supplementary Information

Supplementary Information.

## Data Availability

All data are contained within the manuscript.

## Abbreviations

EVOO: Extra-virgin olive oil
EU: European Union
GPCR: G protein-coupled receptor
TRC: Taste receptor cell
IP_3_: Inositol trisphosphate
DAG: Diacylglycerol
PDE: Phosphodiesterase
HSV: Herpes simplex virus
PROP: 6-N-propylthiouracil

## References

[1] Estruch R (2018). Primary prevention of cardiovascular disease with a mediterranean diet supplemented with extra-virgin olive oil or nuts. N. Engl. J. Med..

[2] Sotos-Prieto M (2019). Assessing validity of self-reported dietary intake within a mediterranean diet cluster randomized controlled trial among US firefighters. Nutrients.

[3] Papanikolaou CME, Magiatis P (2019). Olive oil phenols. Funct. Foods.

[4] Qosa H (2015). Oleocanthal enhances amyloid-beta clearance from the brains of TgSwDI mice and in vitro across a human blood-brain barrier model. ACS Chem. Neurosci..

[5] Pitt J (2009). Alzheimer's-associated Abeta oligomers show altered structure, immunoreactivity and synaptotoxicity with low doses of oleocanthal. Toxicol. Appl. Pharmacol..

[6] Abuznait AH, Qosa H, Busnena BA, El Sayed KA, Kaddoumi A (2013). Olive-oil-derived oleocanthal enhances beta-amyloid clearance as a potential neuroprotective mechanism against Alzheimer's disease: In vitro and in vivo studies. ACS Chem. Neurosci..

[7] Batarseh YS (2017). Oleocanthal ameliorates amyloid-beta oligomers' toxicity on astrocytes and neuronal cells: In vitro studies. Neuroscience.

[8] Monti MC, Margarucci L, Riccio R, Casapullo A (2012). Modulation of tau protein fibrillization by oleocanthal. J. Nat. Prod..

[9] Tsolaki M (2020). A randomized clinical trial of greek high phenolic early harvest extra virgin olive oil in mild cognitive impairment: The MICOIL pilot study. J. Alzheimers Dis..

[10] Elnagar AY, Sylvester PW, El Sayed KA (2011). (-)-Oleocanthal as a c-Met inhibitor for the control of metastatic breast and prostate cancers. Planta Med..

[11] Akl MR (2014). Olive phenolics as c-Met inhibitors: (-)-Oleocanthal attenuates cell proliferation, invasiveness, and tumor growth in breast cancer models. PLoS ONE.

[12] Ayoub NM, Siddique AB, Ebrahim HY, Mohyeldin MM, El Sayed KA (2017). The olive oil phenolic (-)-oleocanthal modulates estrogen receptor expression in luminal breast cancer in vitro and in vivo and synergizes with tamoxifen treatment. Eur. J. Pharmacol..

[13] Filipek A, Czerwinska ME, Kiss AK, Wrzosek M, Naruszewicz M (2015). Oleacein enhances anti-inflammatory activity of human macrophages by increasing CD163 receptor expression. Phytomedicine.

[14] Naruszewicz M, Czerwinska ME, Kiss AK (2015). Oleacein. translation from Mediterranean diet to potential antiatherosclerotic drug. Curr. Pharm. Des..

[15] Nikou T (2019). Comparison survey of EVOO polyphenols and exploration of healthy aging-promoting properties of oleocanthal and oleacein. Food Chem. Toxicol..

[16] Gutierrez-Miranda B (2020). Oleacein attenuates the pathogenesis of experimental autoimmune encephalomyelitis through both antioxidant and anti-inflammatory effects. Antioxidants (Basel).

[17] Busnena BA, Foudah AI, Melancon T, El Sayed KA (2013). Olive secoiridoids and semisynthetic bioisostere analogues for the control of metastatic breast cancer. Bioorg. Med. Chem..

[18] Menendez JA (2008). tabAnti-HER2 (erbB-2) oncogene effects of phenolic compounds directly isolated from commercial Extra-Virgin Olive Oil (EVOO). BMC Cancer.

[19] Leri M (2018). Oleuropein aglycone: A polyphenol with different targets against amyloid toxicity. Biochim. Biophys. Acta Gen. Subj..

[20] Luccarini I (2015). Oleuropein aglycone protects against pyroglutamylated-3 amyloid-ss toxicity: Biochemical, epigenetic and functional correlates. Neurobiol Aging.

[21] in *Commission Regulation (ECC) No 2591/91* (1991).

[22] Cavallo C, Cicia G, Del Giudice T, Sacchi R, Vecchio R (2019). Consumers' perceptions and preferences for bitterness in vegetable foods: The case of extra-virgin olive oil and brassicaceae—A narrative review. Nutrients.

[23] Predieri S, Medoro C, Magli M, Gatti E, Rotondi A (2013). Virgin olive oil sensory properties: Comparing trained panel evaluation and consumer preferences. Food Res. Int..

[24] Beauchamp GK (2005). Phytochemistry: ibuprofen-like activity in extra-virgin olive oil. Nature.

[25] Peyrot des Gachons C (2011). Unusual pungency from extra-virgin olive oil is attributable to restricted spatial expression of the receptor of oleocanthal. J. Neurosci..

[26] Karkoula E, Skantzari A, Melliou E, Magiatis P (2012). Direct measurement of oleocanthal and oleacein levels in olive oil by quantitative (1)H NMR. Establishment of a new index for the characterization of extra virgin olive oils. J. Agric. Food Chem..

[27] Lindemann B (1996). Taste reception. Physiol. Rev..

[28] Margolskee RF (2002). Molecular mechanisms of bitter and sweet taste transduction. J. Biol. Chem..

[29] Nelson G (2001). Mammalian sweet taste receptors. Cell.

[30] Chandrashekar J (2000). T2Rs function as bitter taste receptors. Cell.

[31] Nissim I, Dagan-Wiener A, Niv MY (2017). The taste of toxicity: A quantitative analysis of bitter and toxic molecules. IUBMB Life.

[32] Behrens M, Gu M, Fan S, Huang C, Meyerhof W (2018). Bitter substances from plants used in traditional Chinese medicine exert biased activation of human bitter taste receptors. Chem. Biol. Drug Des..

[33] Lu P, Zhang CH, Lifshitz LM, ZhuGe R (2017). Extraoral bitter taste receptors in health and disease. J. Gen. Physiol..

[34] Wang Q, Liszt KI, Depoortere I (2020). Extra-oral bitter taste receptors: New targets against obesity?. Peptides.

[35] Bloxham CJ, Foster SR, Thomas WG (2020). A bitter taste in your heart. Front. Physiol..

[36] in *No 432/2012 of 16 May 2012* (ed European Commission) (European Commission, Brussels, Belgium, 2012).

[37] Greene TA (2011). Probenecid inhibits the human bitter taste receptor TAS2R16 and suppresses bitter perception of salicin. PLoS ONE.

[38] Behrens M (2004). The human taste receptor hTAS2R14 responds to a variety of different bitter compounds. Biochem. Biophys. Res. Commun..

[39] Matsunami H, Montmayeur JP, Buck LB (2000). A family of candidate taste receptors in human and mouse. Nature.

[40] Brockhoff A, Behrens M, Niv MY, Meyerhof W (2010). Structural requirements of bitter taste receptor activation. Proc. Natl. Acad. Sci. U. S. A..

[41] Born S, Levit A, Niv MY, Meyerhof W, Behrens M (2013). The human bitter taste receptor TAS2R10 is tailored to accommodate numerous diverse ligands. J. Neurosci..

[42] Lossow K (2016). Comprehensive analysis of mouse bitter taste receptors reveals different molecular receptive ranges for orthologous receptors in mice and humans. J. Biol. Chem..

[43] Thalmann S, Behrens M, Meyerhof W (2013). Major haplotypes of the human bitter taste receptor TAS2R41 encode functional receptors for chloramphenicol. Biochem. Biophys. Res. Commun..

[44] Bufe B, Hofmann T, Krautwurst D, Raguse JD, Meyerhof W (2002). The human TAS2R16 receptor mediates bitter taste in response to beta-glucopyranosides. Nat. Genet..

[45] Reichling C, Meyerhof W, Behrens M (2008). Functions of human bitter taste receptors depend on N-glycosylation. J. Neurochem..

[46] Ueda T, Ugawa S, Yamamura H, Imaizumi Y, Shimada S (2003). Functional interaction between T2R taste receptors and G-protein alpha subunits expressed in taste receptor cells. J. Neurosci..

[47] Andreadou I (2006). The olive constituent oleuropein exhibits anti-ischemic, antioxidative, and hypolipidemic effects in anesthetized rabbits. J. Nutr..

[48] Diamantakos P (2015). Oleokoronal and oleomissional: new major phenolic ingredients of extra virgin olive oil. OLIVAE.

[49] Karkoula E, Skantzari A, Melliou E, Magiatis P (2014). Quantitative measurement of major secoiridoid derivatives in olive oil using qNMR. Proof of the artificial formation of aldehydic oleuropein and ligstroside aglycon isomers. J. Agric. Food Chem..

[50] Mousouri E, Melliou E, Magiatis P (2014). Isolation of megaritolactones and other bioactive metabolites from 'megaritiki' table olives and debittering water. J. Agric. Food Chem..

[51] Rigakou A, Diamantakos P, Melliou E, Magiatis P (2019). S-(E)-Elenolide: A new constituent of extra virgin olive oil. J. Sci. Food Agric..

[52] Tsolakou A (2018). Oleocanthalic acid, a chemical marker of olive oil aging and exposure to a high storage temperature with potential neuroprotective activity. J. Agric. Food Chem..

[53] Wang Y (2019). Metal ions activate the human taste receptor TAS2R7. Chem. Senses.

[54] Behrens M (2006). Members of RTP and REEP gene families influence functional bitter taste receptor expression. J. Biol. Chem..

[55] Tsutsui K (2016). Variation in ligand responses of the bitter taste receptors TAS2R1 and TAS2R4 among New World monkeys. BMC Evol. Biol..

[56] Meyerhof W (2010). The molecular receptive ranges of human TAS2R bitter taste receptors. Chem. Senses.

[57] Fotsing JR (2020). Discovery and development of S6821 and S7958 as potent TAS2R8 antagonists. J. Med. Chem..

[58] Sainz E (2007). Functional characterization of human bitter taste receptors. Biochem J.

[59] Kohl S, Behrens M, Dunkel A, Hofmann T, Meyerhof W (2013). Amino acids and peptides activate at least five members of the human bitter taste receptor family. J. Agric. Food Chem..

[60] Grassin-Delyle S (2013). The expression and relaxant effect of bitter taste receptors in human bronchi. Respir. Res..

[61] Dotson CD (2008). Bitter taste receptors influence glucose homeostasis. PLoS ONE.

[62] Li, X., Xu, H., Li, Q., Tang, H. & Pronin, A. Identification of bitter ligands that specifically activate human t2r receptors and related assays for identifying human bitter taste modulators. US 20110136112 A1. (2011).

[63] Thomas A (2017). The bitter taste receptor TAS2R16 achieves high specificity and accommodates diverse glycoside ligands by using a two-faced binding pocket. Sci. Rep..

[64] Duffy VB (2004). Bitter receptor gene (TAS2R38), 6-n-propylthiouracil (PROP) bitterness and alcohol intake. Alcohol. Clin. Exp. Res..

[65] Li, X., Xu, H., Tang, H. & Li, Q. Human T2R receptors for acetaminophen, ranitidine, strychnine and denatonium and related assays for identifying human bitter taste modulators. US8273542B2. (2012).

[66] Brockhoff A (2011). Receptor agonism and antagonism of dietary bitter compounds. J. Neurosci..

[67] Behrens M (2009). The human bitter taste receptor hTAS2R50 is activated by the two natural bitter terpenoids andrographolide and amarogentin. J. Agric. Food Chem..

[68] Chen TW (2013). Ultrasensitive fluorescent proteins for imaging neuronal activity. Nature.

[69] Panizzi LS, Oriente EG (1960). Structure of the bitter glucoside oleuropein. Note II. Gazz. Chim. Ital..

[70] Diamantakos P, Giannara T, Skarkou M, Melliou E, Magiatis P (2020). Influence of harvest time and malaxation conditions on the concentration of individual phenols in extra virgin olive oil related to its healthy properties. Molecules.

[71] Diamantakos, P. *Investigation of the factors affecting the concentration of bioactive phenols in olive oil and methods of their isolation at semi-industrial scale. PhD thesis.* Ph.D. thesis, National and Kapodistrian University of Athens, Greece, (2020).

[72] Koudounas K (2015). A defence-related Olea europaea beta-glucosidase hydrolyses and activates oleuropein into a potent protein cross-linking agent. J. Exp. Bot..

