# Supplementary material for: Activation of specific bitter taste receptors by olive oil phenolics and secoiridoids
Source: Sci Rep. 2021 Nov 16;11:22340. doi: 10.1038/s41598-021-01752-y (PMC8595653; doi:10.1038/s41598-021-01752-y)
Supplement: Supplementary file 1 — Supplementary Information. [file 41598_2021_1752_MOESM1_ESM.pdf]

Activation of specific bitter taste receptors by olive oil phenolics and secoiridoids

Meng Cui[1*], Bohan Chen[1], Keman Xu[1], Aimilia Rigakou[2], Panagiotis Diamantakos[2], Eleni Melliou[2], Diomedes E. Logothetis[1,3,4*] and Prokopios Magiatis[2*]

Supplementary information

[1]Department of Pharmaceutical Sciences, School of Pharmacy, Bouvé College of Health Sciences, Northeastern University, Boston, Massachusetts, 02115, USA

[2] Laboratory of Pharmacognosy and Natural Products Chemistry, Department of Pharmacy, National and Kapodistrian University of Athens, Panepistimiopolis Zografou, 15771 Athens, Greece

[3] Chemistry and Chemical Biology, College of Science, Northeastern University, Boston, MA 02115, USA

[4] Center for Drug Discovery, Northeastern University, Boston, MA 02115, USA

* To whom correspondence should be addressed: m.cui@northeastern.edu (MC); d.logothetis@northeastern.edu (DEL); magiatis@pharm.uoa.gr (PM).

Running title: Olive oil phenolics activate bitter taste receptors

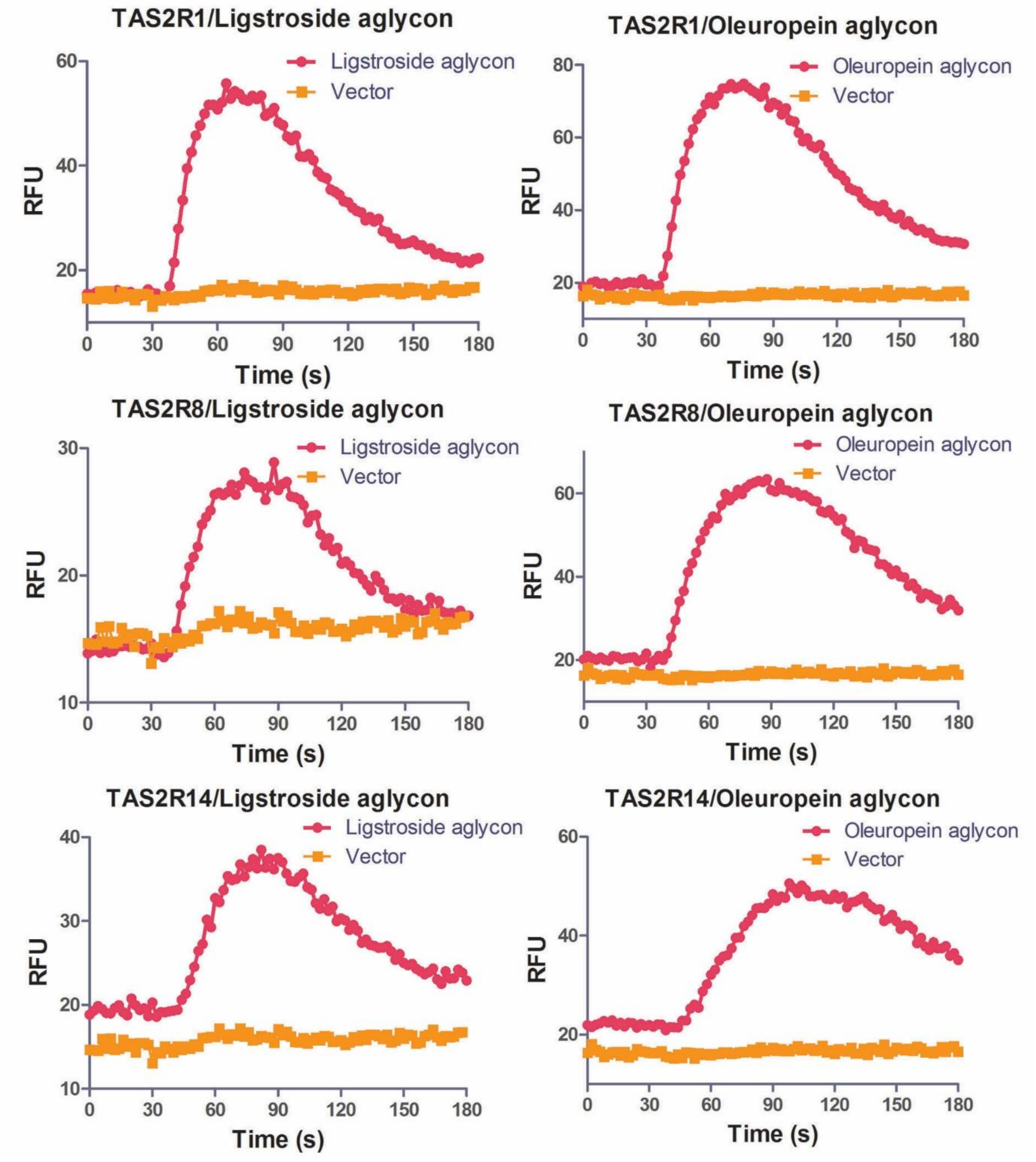
TAS2R1/Ligstroside aglycon
Ligstroside aglycon
Vector
RFU
Time (s)
TAS2R1/Oleuropein aglycon
Oleuropein aglycon
Vector
RFU
Time (s)
TAS2R8/Ligstroside aglycon
Ligstroside aglycon
Vector
RFU
Time (s)
TAS2R8/Oleuropein aglycon
Oleuropein aglycon
Vector
RFU
Time (s)
TAS2R14/Ligstroside aglycon
Ligstroside aglycon
Vector
RFU
Time (s)
TAS2R14/Oleuropein aglycon
Oleuropein aglycon
Vector
RFU
Time (s)

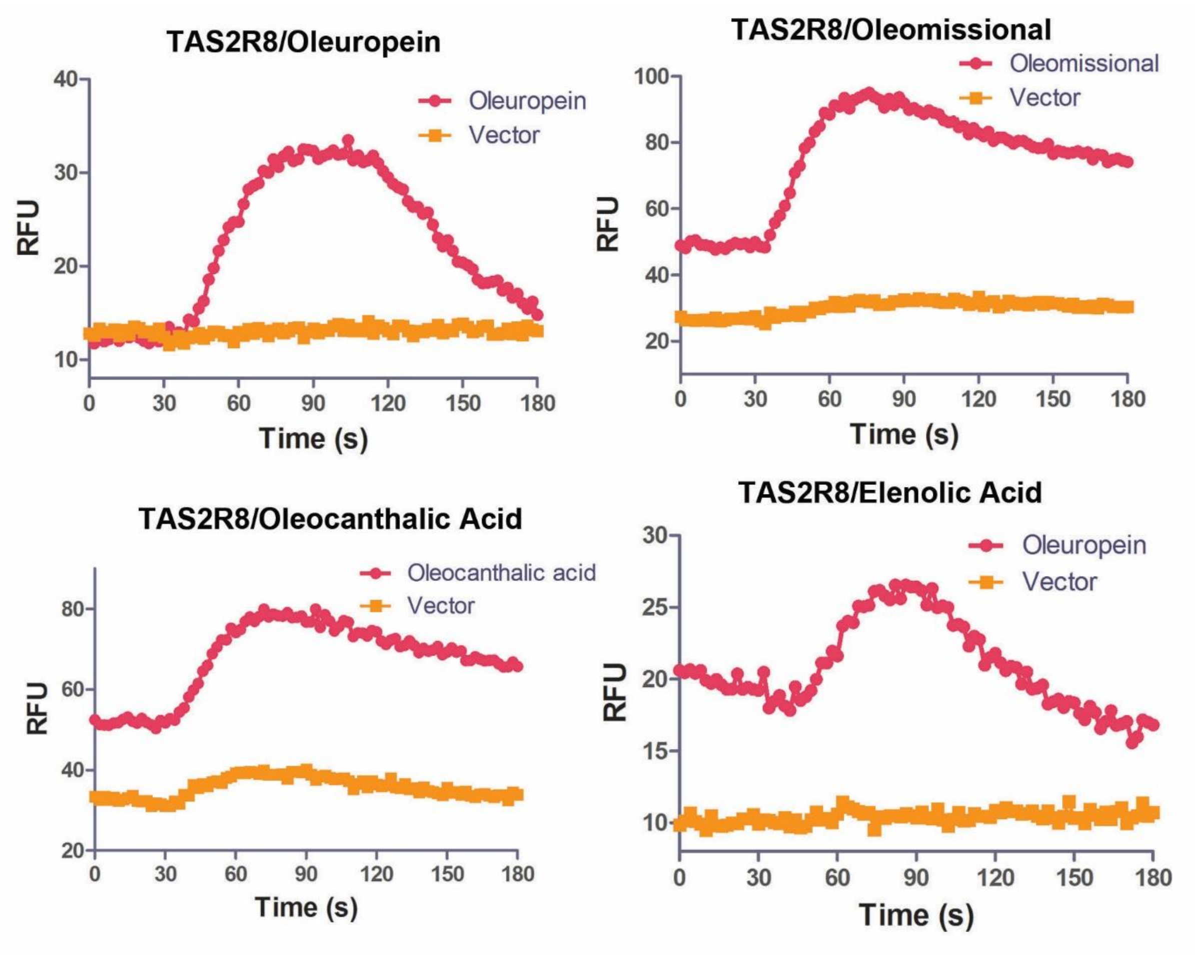

Figure S1. Representative traces from Calcium mobilization assay. Ligstroside aglycon [200µM] activates TAS2R1, TAS2R8, and TAS2R14; Oleuropein aglycon [200µM] activates TAS2R1, TAS2R8, and TAS2R14; Oleuropein [3mM], Oleomissional [300µM], Oleocanthalic acid [300µM], and Elenolic acid [300µM] activate TAS2R8. Compounds were injected at 30s, RFU: relative fluorescence units.
